# Mortality Trends by Causes of Death and Healthcare during a Period of Global Uncertainty (1990–2017)

**DOI:** 10.3390/healthcare9060748

**Published:** 2021-06-18

**Authors:** Simona-Andreea Ursache, Vicentiu-Robert Gabor, Ionel Muntele, Mihai Maftei

**Affiliations:** 1 Faculty of Geography and Geology, Alexandru Ioan Cuza University of Iasi, 700505 Iasi, Romania; gaborvicentiu@gmail.com (V.-R.G.); ionel.muntele@uaic.ro (I.M.); 2 National Institute of Statistics, Iasi County Directorate of Statistics, 700505 Iasi, Romania; 3 Geographical Team, Iasi Branch, Romanian Academy, 700506 Iasi, Romania; 4 German Research Center for Artificial Intelligence (DKFI), Stuhlsatzenhausweg 3, Saarland Informatics Campus D 3_2, 66123 Saarbrücken, Germany; mihai.maftei@dfki.de

**Keywords:** mortality, healthcare challenges, socio-economic impact, world, transition, territorial inequality

## Abstract

In this study we aim to highlight the spatial differences, intensity and frequency of causes of death associated with a range of diseases and the implications of the socio-economic impact on healthcare worldwide between 1990 and 2017: (1) Background: At the same time, an attempt was made to find regional spatial patterns that may be typical for a given geographical area, based on the assumption that global health care is in a permanent state of uncertainty as developed countries have a different morbidity profile than emerging or developing countries. (2) Methods: Using information provided by Global Burden of Disease Collaborative Network, Our World in Data and the World Bank, a multidimensional analysis was carried out, comprising four types of statistical models: grouping analysis, principal component analysis (PCA) Bravais–Pearson linear correlation and multivariate regression. (3) Results: The results confirm the hypothesis of significant correlations between the frequency of causes of death, quality of health care and quality of public health infrastructure, validated by incidence with socio-economic indicators. The study contributes to the literature by analysing trends in the spatial distribution of causes of death worldwide, detecting regional differentiations and testing how socio-economic factors may limit the incidence of morbidity.

## 1. Introduction

Ongoing modernisation and globalisation trends, increasing life expectancy, and decreasing mortality are a predictor of the progress of today’s society and indirectly of well-being. As a result of the uneven demographic transition over time and space, there are still large differences between developed and developing countries, even though in both cases the mortality trend is downwards. On the one hand, there is a lack of medication and modern medical infrastructure in developing countries and, on the other, a diversification of causes of death in industrialised countries. In the case of the latter, there seems to be a steady decline in the rate of deaths caused by diseases of the circulatory system, while the proportion of other causes is rising alarmingly: diseases of the genitourinary system, neoplasms, mental disorders, and diseases of the nervous system. This diversification of morbidity can most likely be attributed to the continuous increase in life expectancy, which has brought new challenges for health systems, such as early diagnosis or awareness of the seriousness of some diseases [1]. In the last century, the reduction of infant and maternal mortality, which in developing countries has led to an explosion of the infectious diseases such as diarrhoea, respiratory diseases, tuberculosis, and malnutrition, considered “the diseases of poverty”. In developed countries, mortality from cancer, heart disease and stroke, known as “diseases of affluence” [2], have gradually been reduced.

This diversity of causes of death worldwide, their frequency and intensity in certain regions, the accessibility of health care, and their differentiated spatial and temporal manifestation were important premises in the design and implementation of this research, which provides a geographical vision, in line with the interest in interdisciplinary research on health status in the current pandemic context. Systematising the typology of causes of death worldwide can provide useful information for current public health policymaking, for the development of health care and infrastructure, and even for understanding the spread of the coronavirus disease 2019 (COVID-19) pandemic, as individual and collective health status, standard of living and access to quality health care can make the difference between life and death.

This study has been structured into six distinct sections as follows: Section 2 comprises a review of the literature, as the foundation of the working hypotheses, considering the main theories and models of the causes of mortality worldwide. Section 3 describes the data used and methodology. This section includes a description of the variables used in the study, their nature, and the statistical models for data analysis: grouping analysis, principal component analysis (PCA), Bravais–Pearson linear correlation and multivariate regression. Section 4 presents the results obtained from the application of the proposed methodology, all four models confirming the correlation between the frequency of causes of death and the socio-economic indicators used. Finally, Section 5 includes discussions, and Section 6 outlines a series of conclusions. The study is based on a specific scientific approach, which is to outline Romania’s position at global level, both in terms of the frequency and intention of some causes of death and to explain a set of peculiarities observed in the doctoral thesis.

## 2. Literature Review

Concerns for the analysis of the spatial diffusion and causality of diseases have been emerging since antiquity (Hippocrates discusses the importance of environmental factors, climate or diet, and their effects on health), were intensified during the Middle Ages against the background of the great epidemics and geographical discoveries, and later, in the modern and contemporary periods, the foundations were laid for disciplines to study the causal relationship between morbidity and other determinants (geographical, social, economic, political etc.) medical geography, health geography, public health, epidemiology, medical economics, etc. The first research studies aimed at developing a general theory of the origin of disease were carried out by the microbiologist René Dubos (1950–1960s), who drew attention to the impotence of determinants in shaping health. Europe has been privileged at the global level because access to detailed statistics on mortality by cause of death has provided the basis for the development of the “epidemiological transition” theory, which faithfully captures, at the micro and macro-territorial levels, the interactions between health patterns, diseases and their determinants, and their economic, social and demographic consequences [3,4], or more concretely “conceptually, the theory focuses on the complex change in patterns of health and disease and on the interactions between these patterns and their demographic, economic and sociologic determinants and consequences” [3,5]. Omran (1971), who formulated the theory, considered that there are three distinct periods through which a steady increase in life expectancy is achieved: “the age of pestilence and famine" (fluctuating increases in mortality), “the age of receding pandemics” (considerable increases in life expectancy) and “the age of degenerative and man-made diseases” (constant decreases in morality, mortality from infectious diseases decreases almost to a minimum while the incidence of degenerative diseases, caused by excess tobacco, alcohol or car accidents, increases) as side effects of modernization [6,7]. Even if it has subsequently been criticised, revised, or adapted, this theory remains an important landmark in demographic and epidemiological studies.

Industrialised countries have moved most rapidly through the stages proposed by Omran’s model, reaching after 1970 what some researchers call “a four-stage”, “cardiovascular revolution” or “the age of delayed degenerative diseases”, with mortality attributable to degenerative diseases occurring in increasingly older populations (Olshansky and Ault, 1986; “the hybristic stage”—Rogers and Hackenberg, 1987) [4,8]. Advanced cardiovascular research, the development of medication and early diagnosis have led to a maximum convergence of life expectancy in the aforementioned countries compared with Eastern European countries, which have not managed to reach the third stage of transition due to the oppressive socio-economic and political context (focused on the centralisation of health care), or with African countries, which have been strongly affected by the emergence of the acquired immune deficiency syndrome (AIDS) epidemic (with inevitable consequences for the precarious local health services), and have thus followed a rather divergent path [9]. As a consequence of the increase in life expectancy, in recent centuries we have also witnessed an increasing spread of chronic and degenerative diseases among rich, emerging and middle-class populations, while the perpetuation of social inequalities, lack of health infrastructure and public health policies have left poor populations increasingly exposed to infectious diseases [10]. In the article “The rise and fall of diseases: reflections on the history of population health in Europe since ca. 1700”, Mackenbach (2020) provides an interesting spatiotemporal summary of the incidence of diseases, explaining that the interaction between people and their environment is at the basis of the spread and subsequent evolution of both infectious diseases (plague, cholera, or syphilis which arose as a result of long-distance travel in Asia and America, tuberculosis, typhoid or diphtheria, which were considered ‘diseases of urbanisation’ as a result of the rural–urban exodus in the 19th century) and chronic and degenerative diseases (the increase in the incidence of various types of cancer as a result of changes in living conditions and human behaviour following the ‘social and economic progress specific to industrialised countries’). It has thus been observed that the incidence of diseases caused by endogenous factors (genetic mutations in the case of Down’s syndrome, Edwards syndrome, DiGeorge syndrome, etc.) is much lower than the incidence of diseases attributed to exogenous causes, with people constantly exposing themselves to health risks in the search for a better life that they did not realise until it was too late [11]. In his book “The Origins of Human Disease” (1991), McKeown (2009) proposes a new theory according to which the origin of all diseases can be classified into only three distinct categories: prenatal diseases (malformations, genetic and chromosomal diseases), diseases of poverty (post-natal: malnutrition, infectious diseases) and diseases of affluence (cardiovascular, cancer, diabetes etc.) [12]. After 1980, in the context of increasing incidence of chronic diseases, changing disease profiles and disability among the elderly, a number of particular models for analysing mortality, morbidity or ageing of the population have been developed, including:

- “the expansion of morbidity hypothesis” (the American epidemiologist Gruenberg (1977) pointed out that medical progress at that time was remarkable and that antibiotics were the most innovative invention in saving lives but nevertheless medical care should not only focus on saving lives, but also on improving the health and disability of the chronically ill because, as a consequence, increasing life expectancy will only lead to an extension of illness and disability, with profound implications for increased public spending on health care; and American mental health biostatistician Morton Kramer has made similar arguments);
- “the compression of morbidity hypothesis” (a model proposed by epidemiologist and rheumatologist Fries (1980), who considered it important to compress mortality around the age of 85, despite the constant improvement in life expectancy, because senility will eventually lead to “natural death”, after which it will be difficult for the body to recover from even the slightest suffering; encouraging the development of preventive medicine);
- “the dynamic equilibrium hypothesis”, (a model proposed by the American demographer Kenneth Manton, as a mediation between the two models mentioned above; based on four distinct principles:
  - “an individual consists of many organs and each of them senesce in their own rate. Thus, the occurrence of deaths depends on the organ that first reaches insufficient levels of capacity”;
  - “these states of component failure might be identified with major chronic degenerative diseases”;
  - “effective prevention or treatment of an individual component failure can postpone death of the organism. Since many diseases share the same risk factors, reduction in disease progression rate within one component might also slow down disease progression within another component”;
  - “every individual experience his own risk of dying, constituting a heterogeneity and selective mortality process that is concealed in average population indicators” [8,13,14].

**Figure 1:**
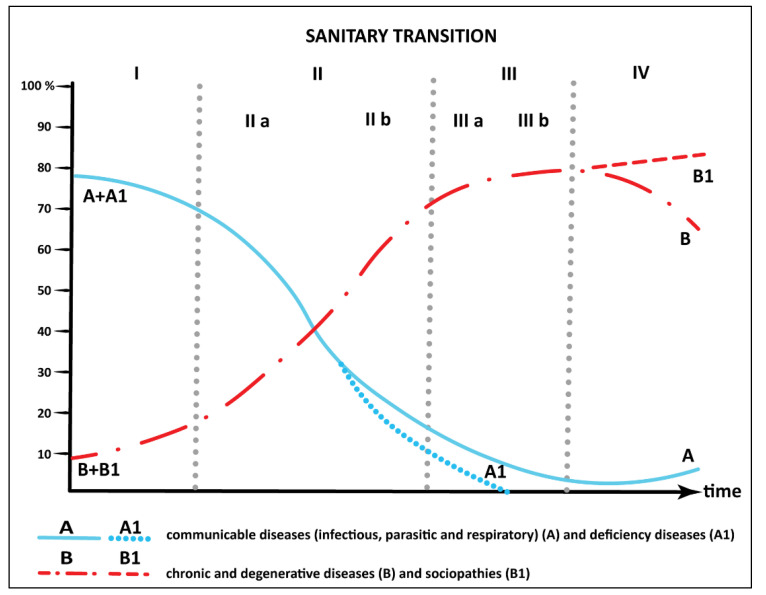
Phases of the epidemiological transition (Omran and Picheral, 1996). Source: Adaptation after: Picheral Henri (1996), La transition sanitaire dans le monde (World health transition).

Omran (1998) added a new stage (alongside the three stages of the epidemiological transition developed in 1971), that of the emergence of new outbreaks of infectious diseases (AIDS, cholera, diphtheria, dengue fever) affecting mainly developing countries, but which should not be ignored by developed countries either [15]. Meslé and Vallin (2002) consider that after the 2000s we can speak of a transition from epidemiological transition to “sanitary transition” (Figure 1), by cause of improvements in public and private hygiene, nutrition and lifestyle, medical techniques, and health services [16,17,18,19]. The term “sanitary transition” was popularized and came into use after the Rockefeller Foundation used this phrase in sanitation programmes (eradication of worms and other types of parasites, hygiene training, construction of latrines, training of health workers in developing countries, etc.). These programmes were promoted because the term epidemiological transition seemed more complex, suggesting the idea of disease rather than health or hygiene [20].

Other authors were of the opinion that the three phases of the epidemiological transition developed by Omran were nothing more than the first phase of a health transition, influenced by medical discoveries, the effective fight against cardiovascular diseases, early diagnosis of cancer, diversification of the public health infrastructure generating greater accessibility to health services, changes in lifestyle or individual behaviour (nutrition, prevention campaigns, immunisation etc.) [21,22]. The concept of health transition has also been used because, after the epidemiological transition, life expectancy has not increased so rapidly in developed countries, even though the overall decline in infectious diseases has meant global progress. The emergence and perpetuation of chronic diseases has brought to light other dimensions of health, with an emphasis on other determinants that were somewhat overlooked in the past: behaviour and lifestyle, physical exertion, alcohol and tobacco consumption, material wealth, level of education etc. [23]. After 1980, there was a virtual reshaping of public health, with the World Health Organisation paying particular attention to the renewal of programmes dedicated to reducing the incidence of tuberculosis and chronic diseases, preventive measures, and the development of public–private partnerships [24,25].

### Proposed Working Hypotheses

**Hypotheses 1** **(H1).** *Healthcare worldwide is in a permanent state of uncertainty as prosperous communities have a different disease profile to disadvantaged ones, with new disease profiles associated with demographic ageing and highly globalised lifestyles creating new inequalities, which historically can be seen as a consequence of progress.*

**Hypotheses 2** **(H2).** *The spatially differentiated distribution, intensity, and frequency of causes of death associated with a range of diseases and the implications of socio-economic impacts on global health care outline highly regionalised spatial structures that could be correlated with the quality of the health system in particular countries.*

**Hypotheses 3** **(H3).** *There is a correlation between the causality of global deaths and a number of socio-economic indicators relevant for the proposed purpose: gross domestic product (GDP), life expectancy, Human Development Index, state fragility, share of obese population, share of government medical expenditure, etc.*

## 3. Materials and Methods

### 3.1. Data Collection

The data collected for the analysis were obtained from several internationally accredited statistical sources:

- IHME (Institute for Health Metrics and Evaluation) [26], the Global Burden of Disease Collaborative Network, is an independent population health research institute at UW Medicine, part of the University of Washington, that provides health-related statistics, but also with expertise in other related areas, such as the causation of violent deaths (suicide, terrorism, road traffic accidents etc.) The Global Burden of Disease is a major global study on the causes of death and disease published in the medical journal *The Lancet* [27]. The resource has been the primary source for disseminating global data on deaths by cause. We considered data from 1990–2017 (27 years), and the top 14 global killer disease types (threshold of 500,000 deaths in 2017), with malaria being the last condition considered (Table 1). In order to obtain the most accurate database possible, the variables were standardised by group maximum. The study was based on data from 194 countries, reported per 100,000 inhabitants, averaged over 27 years of study.

Non-communicable diseases, often chronic and long-lasting, dominate global mortality figures (the leading cause: cardiovascular deaths dominating the entire hierarchy with very high values: 17.79 million deaths in 2017; followed by cancer deaths: 9.56 million deaths in 2017; and deaths from respiratory diseases: 3.91 million in 2017), affecting mainly developed countries. At the opposite pole are “natural deaths” (9602 deaths in 2017), deaths due to terrorism (26,445 in 2017) and exposure to heat (or cold: 53,349 deaths in 2017), with deaths due to infectious diseases, malnutrition, maternal and neonatal deaths being specific to low-income countries with low living standards [27].

- OWID (Our World in Data), an institute, but also an online scientific publication, which generally focuses on the study of global social issues. The institute does not generate its own statistical data but mention of this institute is necessary because the initial data was obtained through this “portal” [28].
- World Bank, the most important institution in the collection of statistical data worldwide [29]. The data on social and economic aspects of society (the exploratory variables) were obtained from this institution. The organisation collects statistical data from all spheres of society and beyond. The purpose of the proposed variables is to highlight socio-economic impact, health care and to help outline typical territorial structures with a relatively uniform distribution (Table 2).

### 3.2. Data Analysis Methods

From a methodological point of view, in order to test the proposed analyses, four main analysis models were operated, namely: multivariate regression, Bravais–Pearson linear correlation, principal component analysis (PCA) and the grouping analysis method performed in ArcMap 10.6. The Bravais–Pearson multivariate correlation, which is “one of the most widely used statistical methods to eliminate trivial information” (i.e., those characterizing the whole space under investigation) was operated in XLSTAT Version 2014.5.03 Copyright Addinsoft 1995–2014 (2014) XLSTAT and Addinsoft are Registered Trademarks of Addinsoft, https://www.xlstat.com (accessed on 17 June 2021) [30,31].

The four analyses carried out attempt to capture in as much detail as possible the links between the causalities resulting from the main diseases worldwide and a series of socio-economic particularities and, at the same time, a geographical approach to the topic was attempted by studying the phenomena from the perspective of geographical space.

The grouping analysis method was carried out with the ArcMap software. 10.6, from ESRI (Environmental Systems Research Institute). One of the ArcMap tools software can identify a series of characteristics that show the existence of similarities within a group, in this case the similarities between the countries of the world in terms of the theme addressed in our study. The clustering results of this tool are based on the number of clusters specified, the analysis variables and optional spatial constraints. Performing several iterations serves to identify an optimal number of clusters as well as an appropriate combination of analysis variables [32].

In the present case, 14 conclusive variables were used, which aimed to result in worldwide typologies (194 countries) of the manifestation of the main diseases in territorial profile. No spatial constraint was used for the analysis, the distance used in the analysis was Euclidean. All the variables of the resulting analysis were standardised according to the group maximum. The role of the standardisation was to bring the values to a common denominator, to result in statistically comparable data sets. Four classes were selected for presenting the results of the grouping analysis, the reason for the selection being the usefulness of presenting the world as conclusively as possible without complicating the analysis with too many groupings of data.

Two geostatistical products were obtained from the analysis, a cartogram with the corresponding typologies, showing how the world states were divided into spatialised groups. The second product consisted of a visual summary for the interpretation of the four typologies obtained. The result presented visually in the form of statistical boxplots for each variable. The “Grouping Analysis” tried to provide a spatial overview of the main causes of death according to the diseases present worldwide, a strongly statistically based perspective, and at the same time, an attempt was made to synthesize the presence of similarities between the countries of the world in the manifestation of the causalities studied, an analysis similar to that of statistical clusters. PCA is a way to reduce large datasets, aiming to facilitate interpretation with minimal information loss. Reducing the dataset size is an important step for visualizing and processing large data series [33]. PCA analysis is a method to simplify statistical correlations visually, facilitating a quick interpretation of analysis and having an impact within academia. Pearson-B Correlation, and multivariate regression presented in the form of correlation tables, were performed precisely so that the picture between deaths from major diseases worldwide and a number of exploratory socio-economic variables could be as detailed and complete as possible.

**Figure 2:**
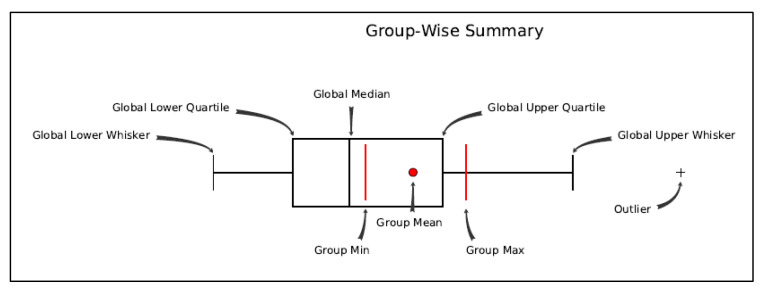
Methodological bloxplot. Source: GIS Methodology Esri.com, resulted with ArcMap 10.6.

**Figure 3:**
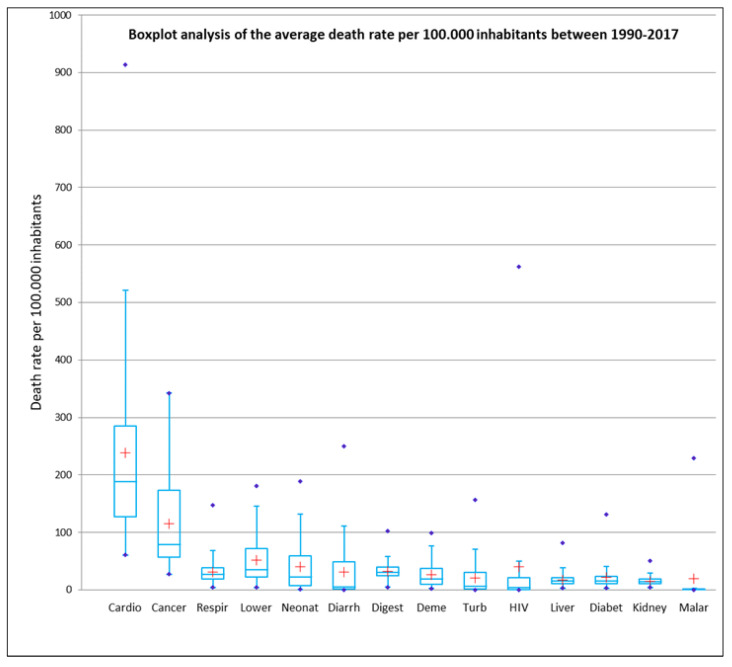
Representative Boxplot analysis for data interpretation. Source of data: The Institute for Health Metrics and Evaluation, World Bank.

The boxplot representation (Figure 2) frames the most essential statistical features of a frequency distribution to have a better understanding and the possibility to perform a comparative analysis (Figure 3). The boxplot visualization is based on the synthesis of the five displays (“minimum”, first quartile (Q1), median, third quartile (Q3) and “maximum”), [34]. Boxplot analysis captures the intensity of deaths resulting from major diseases worldwide, the impact of diseases in the global hierarchy, but also the disparities between countries in the world on this issue, observable by comparing the large differences between the mean and median values for each variable.

## 4. Results

**Figure 4:**
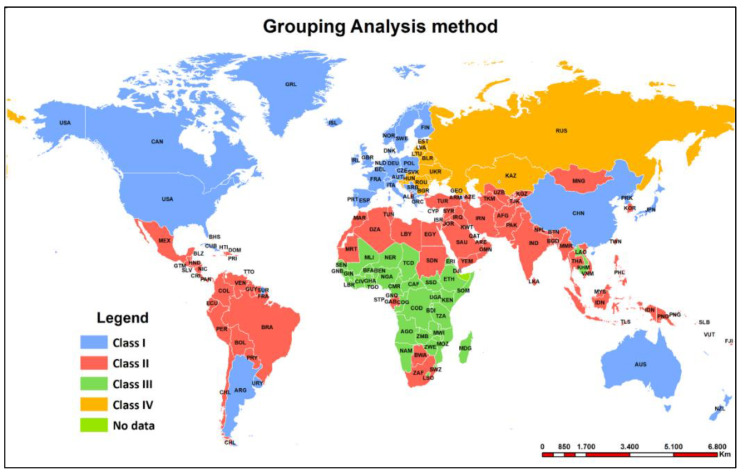
Global distribution of mortality causes by the grouping analysis methods. Source of data: The Institute for Health Metrics and Evaluation, World Bank.

The proposed study aims to highlight the overall trends in mortality by cause of death, the socio-economic implications, and the uncertainty generated by health care between 1990 and 2017 (Figure 4). In order to generate the analysis of the causes of death worldwide as comprehensive as possible, we have considered it appropriate to include some indicators less used in the literature in this formula. The triad of economic–social–health indicators linked to the 14 major causes of death worldwide have outlined a very interesting and timely global profile in the current pandemic context. By using the grouping analysis method, a spatial overview of the main causes of death according to the diseases present worldwide was obtained, by showing similarities between countries in the manifestation of the causes studied, an analysis which confirmed the hypotheses proposed. The results of PCA summarised the correlations obtained, in a simplified presentation, to make it easier to validate the hypotheses.

**Figure 5:**
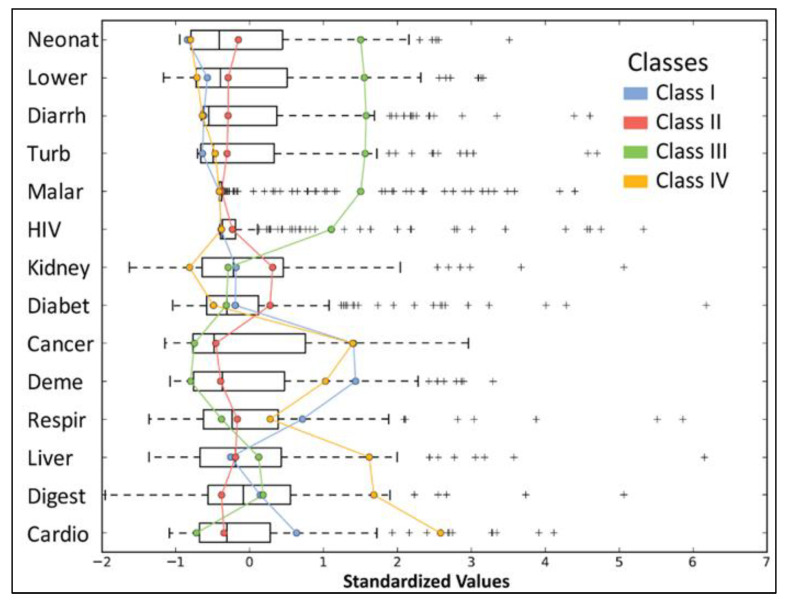
Standardized values by the grouping analysis methods. Source of data: The Institute for Health Metrics and Evaluation, World Bank.

The chart above (Figure 5) shows the intensity and frequency of deaths resulting from the diseases analysed. The larger the interquartile space, the more evenly they tend to be distributed in geographical profile, a situation best reflected by cardiovascular diseases. When the median and mean have close values, the distribution of values tends to be similar. In order to study further the main diseases worldwide, specific Geographic Information Systems (GIS) techniques were used. Using the grouping analysis model, a synthetic typological classification of the world’s countries was obtained, the four classes each having their own particularities, depending on the variables inserted in the analysis. States were grouped according to a number of similarities into the following classes:

Class I, generally Western countries, or countries with historical links to the European continent. The typology shows extremely low values for deaths due to conditions such as neonatal conditions, diarrhoeal diseases, liver diseases, tuberculosis, human immunodeficiency virus (HIV), malaria. At the other end of the scale, there are high values for deaths due to: dementia (countries in this typology are also the most affected areas in the world by this disease), followed by deaths due to cancer, respiratory diseases (respiratory arrest) and cardiovascular diseases. These diseases, which are considered to be “diseases of medical progress”, have a certain cyclical nature over time, even though their area of spread is now much greater. For example, some studies have shown that ischaemic heart disease is not really a “new” disease, with evidence of its existence in much earlier times being found in Egyptian mummies, even though it was considered a rare disease compared to the 20th century [11]. Survival rates have gradually increased in Northern and Western Europe and for certain cancers (thyroid cancer, Hodgkin’s disease, uterine body cancer, malignant melanoma, and testicular cancer) as result of prophylaxis and the effectiveness of new treatments in case of early diagnosis [35]. At the same time, cancer incidence is sensitive to age structure in Western countries, with cancer death rates being higher among older people [36].

China’s placement in this class may seem unexpected, as for some variables we identify high frequencies of occurrence of conditions specific to developing countries, but the placement in this typology was “helped” by the high rate of deaths from cardiovascular conditions, deaths from cancer-related conditions (specific to developed countries), low rate of deaths from neonatal conditions and low rate of deaths from diarrhoeal conditions (specific to developing countries). China’s place alongside developed countries may be an element of originality in the study.

**Figure 6:**
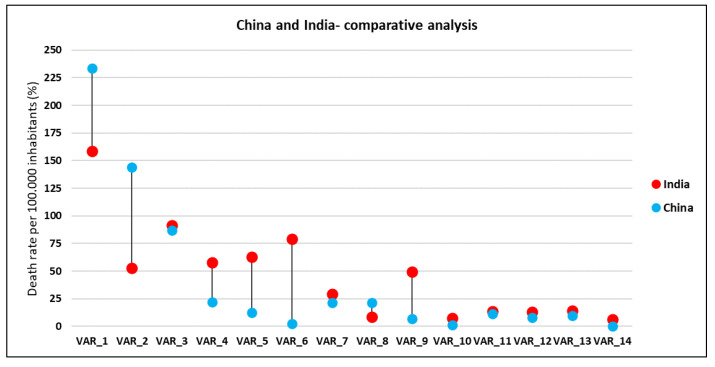
Comparison of the death rate per 100.000 inhabitants between China and India by cause of death (average 1990–2017). Source of data: The Institute for Health Metrics and Evaluation, World Bank.

For characteristics more common to Western Europe and to the countries that have been classified as Class I in the analysis, we propose the figure below, which provides a comparative picture between two countries classified in two distinct typologies in terms of profile, India and China, the most populated countries in the world (Figure 6). On the whole, the diseases in this class are more related to pathologies associated with old age and less to the quality of life issues. China has made remarkable progress in recent decades, with about 80% of deaths caused by chronic diseases and high exposure to risk factors (high male tobacco consumption, a significant proportion of children aged 7–17 in large cities are overweight or obese) [37,38]. An interesting aspect is also the relationship between globalisation and government spending on health or quality of life, with countries with highly globalised economies (such as China) clustering with developed Western countries [39], which may be an original and novel element of the proposed study.

Class II has low values for deaths due to kidney disease, respiratory infections, deaths associated with diabetes and HIV-related conditions. Most countries in this class fell in Latin America, the Middle East and much of the Far East. In the case of Latin America, Frenk et al. formulated the “protracted polarised transition model”, a new conceptualisation designed to explain the process of epidemiological polarisation in these countries, characterised by an overlap of eras. They have experienced the re-emergence of malaria, dengue fever and other infectious diseases combined with the emergence of cancer and other endemic diseases against a background of increasing social and geographical inequalities [40,41]. North Africa and the Middle East have experienced a similar transition, social implications, links to the European continent and substantial public health measures have succeeded in reducing infectious diseases [42]. Class III generally groups countries with deaths due to quality of life conditions. Most of the countries in this group are located in sub-Saharan Africa plus two Asian countries (Cambodia and Laos). They have a number of indicators that point to serious problems in the national health system, exemplified by conditions such as neonatal diseases, diarrhoeal diseases, tuberculosis, malaria and HIV AIDS. Malaria affects mainly areas of East Africa, South and South-East Asia and even South America, and is an epidemic disease that also severely affects pregnant women, leading to premature births or intrauterine growth retardation [43,44]. In the case of AIDS, the territorial distribution is very uneven, with the most affected regions being Southern and Eastern Africa, with slight stagnation or some modest decline in the incidence of the disease being evidenced in Northern and Western Africa. AIDS in these regions also appears to be strongly influenced by migration, male circumcision, and herpes simplex virus infection. In the case of Cambodia and Laos, factors related to intravenous drug use and sex worker networks appear to have a direct influence on AIDS incidence [45,46]. A worrying public health problem in these countries is the association of AIDS, a disease that affects the immune system, with other infectious diseases (malaria, tuberculosis, etc.), leading to an increase in the number of deaths [47,48]. In recent years, however, both prevention and treatment programmes have proved effective, with infected people receiving modern medical services, appropriate treatment and counselling. In Africa morbidity and indirectly epidemiological transition has also been influenced by frequent wars or other forms of political violence. At the opposite pole are deaths caused by diseases specific to Western countries with high life expectancy: dementia, cardiovascular and respiratory diseases. In the countries of this category, health inequalities are also determined by a better understanding of life, more efficient health systems and more resources invested in the determinants of health (sanitation, healthy housing, access to education), with direct implications also in the increase in life expectancy, which still shows very large differences globally (84 years in Japan and Hong Kong compared to Sierra Leone or Central African Republic—52 years) [49]. Another study correlates increased life expectancy with the consumption of medicines, decreased alcohol consumption, tobacco consumption or increased consumption of fruit, vegetables [50].

The last class grouped Eastern European countries, including most of the former Soviet states or some former Yugoslav states (Slovenia and Croatia). The highest incidence here is for cardiovascular, digestive and liver diseases. Cardiovascular diseases have the highest incidence worldwide and can be a predictor of death due to ageing. Cardiovascular diseases are causing increasing territorial inequalities in Europe, as in 2011, for example, the “cardiovascular death rate in Ukraine (the highest) was seven-fold higher than in France (the lowest)” [51]. As the correlation of indicators shows, the incidence of cardiovascular deaths is more specific in countries with a higher median age, demographically ageing countries. Eastern Europe has long lagged behind North-Western Europe in terms of overall quality of life and medical progress, mainly due to “economic centralization”, lack of prosperity being an important consequence for healthy living and indirectly for the development of medical infrastructure [11,52,53]. In the case of Russia, the incidence of cardiovascular disease is also blamed on excessive alcohol consumption leading to arrhythmias and ischemic heart disease or even respiratory diseases and pneumonia related to the continental climatic context [54,55]. Countries in this category have low values for deaths due to kidney disease, respiratory infections, deaths associated with diabetes, or HIV infection.

In conclusion, the results of the grouping analysis summarised the countries of the world in four typological profiles, which summarise regional similarities and differences.

**Figure 7:**
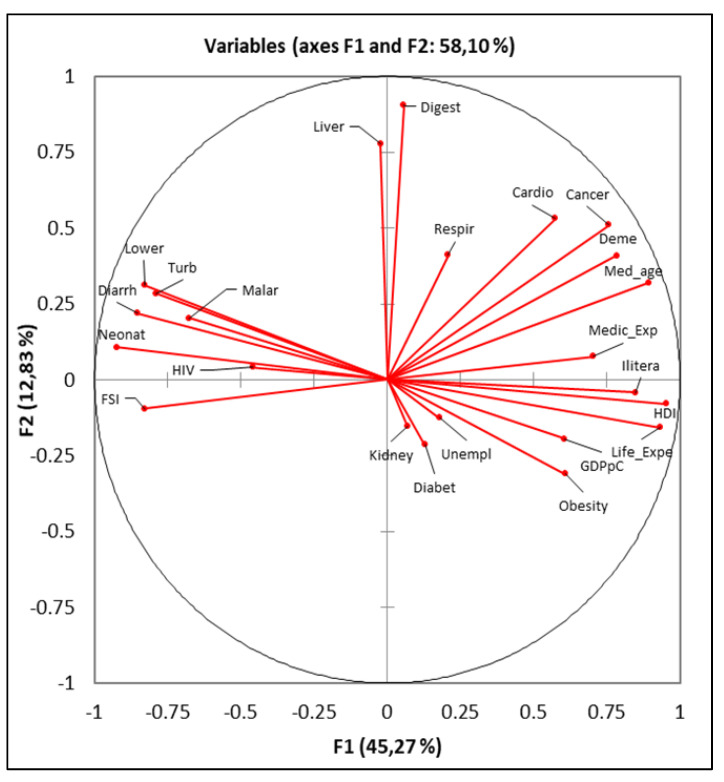
Principal component analysis (PCA) results. Source of data: The Institute for Health Metrics and Evaluation, World Bank.

The advantage of using PCA is that it simplifies the data and makes it possible to synthetically interpret data on the distribution of the main forms of morbidity worldwide and how they are related. The resulting analysis clearly captures a clustering of variables according to a well-defined socio-economic profile. Variables in the F2 coordinate reflect the majority of diseases specific to developing countries, resulting from systemic problems, major economic and social deficiencies (Figure 7).

Principal component analysis indicates that almost all variables are significantly correlated with each other, some more strongly than others. The point cloud obtained from the analysis represents the countries of the world, which have been grouped according to a number of similarities in the characteristics of deaths due to common conditions. The grouping is analogous to the formation of clusters of countries grouped at regional level. States in Europe were positioned in a cluster of points, best highlighted in the figure above. All countries in the world have clustered at a regional level, which means that some diseases have a clear regional pattern. The most common cause of death worldwide, cardiovascular disease, shows a high degree of correlation with cancer death rates, dementia deaths, digestive diseases, and liver diseases. All of these are generally characteristic of the developed world.

**Figure 8:**
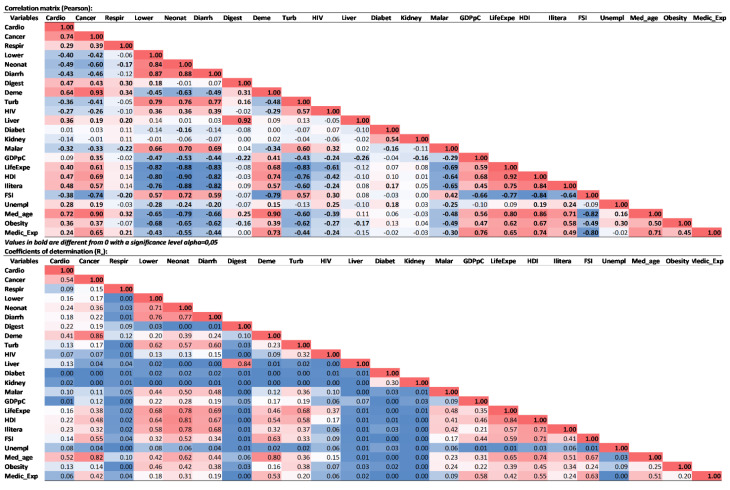
Bravais–Pearson linear correlation and linear regression. Source of data: The Institute for Health Metrics and Evaluation, World Bank.

Negative correlations are present for deaths from conditions such as lower respiratory tract infections, neonatal conditions, diarrhoeal conditions, tuberculosis, HIV, and malaria. The situation is also confirmed by the socio-economic variables, the rate of deaths due to cardiovascular diseases shows a high degree of correlation with: life expectancy, median age of the population, human development index and literate population. At the opposite pole there is a negative correlation with the state fragility index (“The Organization for Economic Cooperation and Development (OECD) characterises fragility as the combination of exposure to risk and insufficient coping capacity of the state, systems and/or communities to manage, absorb or mitigate those risks. Fragility can lead to negative outcomes including violence, poverty, inequality, displacement, and environmental and political degradation”), which implies that deaths from cardiovascular disease are more specific to stable states [56]. Cancer deaths are highly correlated with the variable expressing dementia deaths. At the same time, this disease is more common in countries with a high standard of living, a situation reflected in the variables that capture quality of life: life expectancy, literacy, investment in the medical system, etc. Regardless of how the correlations of the analysis are interpreted, they are captured sufficiently clearly and validate the proposed hypotheses, as death rates from certain diseases have a pronounced regional specificity. Many of them show a pathological profile correlated with the standard of living and level of development of the countries analysed. The analysis also showed confirmation of Pearson correlations and by correlation coefficients R (Figure 8). In cases where there is a discrepancy in identifying links between variables, a detailed case study could be carried out.

## 5. Discussion

The results obtained validated the hypotheses put forward, both in terms of differences between developed and developing countries and the manifestation of strongly regionalised spatial structures, possibly related to the quality of the health system. As regards the causal relationship with the socio-economic indicators considered relevant, it was not fully certified, showing a much stronger specificity at national level. This has also produced apparent “anomalies” such as the association of China in the same group (cluster) with developed countries or of Laos and Cambodia with sub-Saharan African countries. The distribution of specific causes of death confirms the theoretical patterns of analysis reviewed in the introductory part, with the epidemiological transition and the newer, health-related transition being unquestionable. Advanced processing of the information in the database using multivariate statistics allowed significant correlations to be established between the dependent variable and the exploratory variables. The grouping of certain specific causes of death corresponds to a large extent to the descriptive analysis which suggests the weight of the economic and social development factor. Basically, the four classes separated by grouping analysis are quite coincident with the distribution of states along the two axes, F1 and F2, of the PCA. Obviously, the presence of exceptional cases, which deviate from the main group, may raise discussion, as in the case of the Republic of Moldova or Hungary within the Eastern European countries. Romania’s position within the same group, close to Ukraine, at a significant distance from the more compact group of the other Eastern European countries, may also raise discussion, with some studies indicating a strong regional specificity at national level, closely linked to the level of development, the quality of health infrastructure or the particularities of the natural environment [57]. As the patterns of evolution of the causes of mortality at the global level have been highlighted, it can be concluded that they are the expression of distinct phases of the transition of public health systems, from a focus on prevention, typical of developed countries, to efforts to limit forms of morbidity considered to be a thing of the past but still common in developing countries. Eastern European, Latin American, North African, South, and South-West Asian countries can be seen as in transition towards the desirable model of developed countries.

The significance of the grouping of states into the four classes highlighted by the analysis could be used in testing the specific evolution of the recent COVID-19 pandemic. Empirically, based on current information, there seems to be a sufficiently large overlap, with Eastern European countries, for example showing a different evolution than Western countries, including in terms of mortality following this pandemic, as recent studies have shown [58]. The evolution of African, Latin American, and Asian countries also seems to be well documented, except China. The database processed in this study could support a test of how the countries of the world have responded to this challenge. The fact that developed countries have generally, at least so far, been more heavily affected may indicate a greater vulnerability due to the specificity of morbidity dominated by diseases characteristic of older people, the differences between them being determined by the response of the health infrastructure.

## 6. Conclusions

The study aimed to analyse trends in the evolution and spatial distribution of causes of death worldwide, with the objective of detecting regional differences and the existence of specific patterns correlated with socio-economic variables. Beyond certifying the validity of the theoretic models of death causality analysis, the results presented, particularly, the multivariate analysis, demonstrate the complexity of the relationships between the public health system and the variables that may favour the manifestation of some specific causes of death. This may suggest that the epidemiological transition and the health transition are, as the name suggests, transitory processes, lagged in time and space, which are far from constituting a definitive framework in the struggle for a long, healthy life. The current context, marked by the COVID-19 pandemic, could be a turning point capable of modifying the incidence of certain factors, introducing new variables, bringing to light new vulnerabilities, possibly to be used in the construction of a new explanatory model, adapted to a world marked by unprecedented global changes, still insufficiently assumed. The present study can thus also be seen as an exercise in testing the extent to which the determinants of the population’s state of health, albeit viewed from a cynical perspective, are in constant competition with each other, with the aim of limiting the incidence of causes of death that can be controlled by advances in knowledge. The results of the study can thus be used to complement previous research or to support the detailing of analyses at national level. The main contribution of this work is twofold. Firstly, the causes of death are simultaneously correlated with economic indicators (GDP, total unemployment), social indicators (life expectancy, Human Development Index, literate population (%), state fragility index) and healthcare indicators (obese population, medical expenses per person). There are currently few studies that address the issue at the global level from the perspective of the proposed indicators. Secondly, the confirmation of the working hypotheses may represent an interesting premise for development at the national level in the field of public health, by highlighting the main regional cleavages and disparities. The study is also based on a specific scientific approach, that of outlining Romania’s position at global level, both in terms of the frequency and intention of certain causes of death and, in order to explain a series of particularities observed in the doctoral thesis, thus providing the basis for a series of studies at national level (infant mortality, premature mortality, etc.).

## Figures and Tables

**Table 1:** Number of deaths by cause in the world (Source: Institute for Health Metrics and Evaluation, IHME).

	Number of Deaths by Cause	(Dependent Variables)
Cardio—Average rates per 100.000 inhabitants between 1990 and 2017 for deaths from cardiovascular disease. Cancer—Average rates per 100.000 inhabitants between 1990 and 2017 for deaths from cancer. Respir—Average rates per 100.000 inhabitants between 1990 and 2017 for deaths from respiratory diseases. Lower—Average rates per 100.000 inhabitants between 1990 and 2017 for deaths from lower respiratory infections. Neonat—Average rates per 100.000 inhabitants between 1990 and 2017 for deaths from neonatal disorders. Diarrh—Average rates per 100.000 inhabitants between 1990 and 2017 for deaths from diarrheal diseases. Digest—Average rates per 100.000 inhabitants between 1990 and 2017 for deaths from digestive diseases. Deme—Average rates per 100.000 inhabitants between 1990 and 2017 for deaths from dementia. Turb—Average rates per 100.000 inhabitants between 1990 and 2017 for deaths from tuberculosis. HIV—Average rates per 100.000 inhabitants between 1990 and 2017 for deaths from HIV AIDS. Liver—Average rates per 100.000 inhabitants between 1990 and 2017 for deaths from liver diseases. Diabet—Average rates per 100.000 inhabitants between 1990 and 2017 for deaths from diabetes. Kidney—Average rates per 100.000 inhabitants between 1990 and 2017 for deaths from kidney disease. Malar—Average rates per 100.000 inhabitants between 1990 and 2017 for deaths from malaria.		

**Table 2:** Socio-economic variables (Source: World Bank).

Socio-Economic Variables (Exploratory Variables)
GDPpC—Average GDP/per capita in the period 1990–2017. LifeExpe—Average life expectancy in the 1990–2017. HDI—Average Human Development Index (HDI) 1990–2017. Litera—The average share (%) of the literate population in the period 1990–2015. FSI—The average of the state fragility index in the period 2007–2017. Unempl—Total unemployment (%-of total labour force), average in the period 1990–2017 Med_age—Median age (average in the period 1990–2015). Obesity—Share (%) of the obese population, average in the period 1990–2016. Medic_exp—Average medical expenses per person in the period 1995–2017.

## Data Availability

Not applicable.

## References

[1] Bergeron-Boucher M.P., Aburto J.M., Van Raalte A. (2020). Diversification in causes of death in low-mortality countries: Emerging patterns and implications. BMJ Global Health.

[2] Deaton A. (2017). Marea evadare. Sănătatea, Bogăția și Originile Inegalității.

[3] Abdel-Rahmin O. (2005). The epidemiologic transition: A theory of the epidemiology of population change. Milbank Q..

[4] Abdel-Rahmin O. (2001). The epidemiologic transition: A theory of the epidemiology of population change/Abdel R. Omran. Bull. World Health Organ. Int. J. Public Health.

[5] Mc Keown R.E. (2009). The Epidemiologic Transition: Changing Patterns of Mortality and Population Dynamics. Am. J. Lifestyle Med..

[6] Caselli G., Meslé F., Vallin J. (2002). Epidemiologic Transition Theory Exceptions. Genus JSTOR.

[7] Weisz G., Olszynko-Gryn J. (2010). The theory of epidemiologic transition: The origins of a citation classic. J. Hist. Med. Allied. Sci..

[8] Moe J.O., Hagen T. (2011). Trends and variation in mild disability and functional limitations among older adults in Norway, 1986–2008. Eur. J. Ageing.

[9] Vallin J., Meslé F. (2004). Convergences and divergences in mortality. A new approach to health transition. Demogr. Res. Spec. Collect..

[10] Armelagos G.J., Brown P.J., Turner B. (2005). Evolutionary, historical and political economic perspectives on health and disease. Soc. Sci. Med..

[11] Mackenbach J.P. (2021). The rise and fall of diseases: Reflections on the history of population health in Europe since ca. Eur. J. Epidemiol..

[12] Mackenbach J.P. (2020). Chapter 3 Understanding Trends in Population Health. A History of Population Health.

[13] Fries J.F. (1980). Aging, natural death, and the compression of morbidity. N. Engl. J. Med..

[14] Olshansky S.J., Wilkins R. (1998). Introduction. J. Aging Health.

[15] Lussier M., Bourbeau R., Choinière R. (2008). Does the recent evolution of Canadian mortality agree with the epidemiologic transition theory. Demogr. Res..

[16] Meslé F., Vallin J., Caselli G., Vallin J., Wunsch G. (2002). La Transition Sanitaire: Tendances et Perspectives. Démographie: Analyse et Synthèse, III. Les Déterminants de la Mortalité.

[17] Meslé F. (2004). Ecart d’espérance de vie entre les sexes: Les raisons du recul de l’avantage féminin. Rev. d’Epidémiologie St. Publique..

[18] Vallin J., Jasilionis D., Meslé F. (2008). La transition sanitaire à l’épreuve d’une histoire tourmentée: Le cas des pays baltes. Popul. Sociétés..

[19] Vallin J., Meslé F., Tabutin D., Masquelier B. (2010). De la Transition Épidémiologique à la Transition Sanitaire: L’improbable Convergence générale. Ralentissements, Résistances et Ruptures dans les Transitions Démographiques, Actes de la Chaire Quetelet.

[20] Caldwell J.C. (1993). Health transition: The cultural, social and behavioural determinants of health in the Third World. Soc. Sci. Med..

[21] Picheral H. (1996). La transition sanitaire dans le monde (World health transition). Bull. l’Association Géogr. Fr..

[22] Caselli G. (1989). Transition sanitaire et structure par cause de la mortalité: Anciennes et nouvelles causes. Ann. Démo. Hist..

[23] Meslé F., Vallin J. (2000). Transition sanitaire: Tendances et perspectives. M S Med. Sci..

[24] Gaudillière J.P. (2016). Un nouvel ordre sanitaire international? Performance, néolibéralisme et outils du gouvernement médico-économique. Écol. Polit..

[25] Buse K., Harmer A. (2007). Seven Habits of Highly Effective Global Public-Private Health Partnerships. Pract. Potential Soc. Sci. Med..

[26] The Institute for Health Metrics and Evaluation. http://www.healthdata.org/.

[27] Ritchie H., Roser M. (2018). Causes of Death. https://ourworldindata.org/causes-of-death.

[28] Our World in Data. https://ourworldindata.org/.

[29] World Bank. https://www.worldbank.org/en/home.

[30] XLSTAT Version 2014.5.03 Copyright Addinsoft 1995–2014 (2014) XLSTAT and Addinsoft are Registered Trademarks of Addinsoft. https://www.xlstat.com.

[31] Groza O. (2002). SIG între structurile spaţiale generale şi specificul local, Analele Ştiinţifice ale Universităţii “Al. I. Cuza” din Iaşi, (serie nouă), tomul XLVII, s. II c. Geogr. Supl. Lucr. Simp. SIG.

[32] Bonilla C. (2016). Defining Communities with ESRI’s Grouping Analysis Tool, Azavea (blog). https://www.azavea.com/blog/2016/10/12/defining-communities-grouping-analysis-tool/.

[33] Jolliffe I.T., Cadima J. (2016). Principal component analysis: A review and recent developments. Phil. Trans. R. Soc. A.

[34] Tukey J.W. (1977). Tukey, Exploratory Data Analysis.

[35] Mackenbach J.P. (2020). Chapter 2 Long-term Trends in Population Health. A History of Population Health.

[36] Hashim D., Boffetta P., La Vecchia C., Rota M., Bertuccio P., Malvezzi M., Negri E. (2016). The global decrease in cancer mortality: Trends and disparities. Ann. Oncol. Off. J. Eur. Soc. Med. Oncol..

[37] Wang L., Kong L., Wu F., Bai Y., Burton R. (2005). Preventing chronic diseases in China. Lancet.

[38] Yang G., Wang Y., Zeng Y., Gao G.F., Liang X., Zhou M., Wan X., Yu S., Jiang Y., Naghavi M. (2013). Rapid health transition in China, 1990-2010: Findings from the Global Burden of Disease Study 2010. Lancet.

[39] Martín Cervantes P.A., Rueda López N., Cruz Rambaud S. (2020). The Effect of Globalization on Economic Development Indicators: An Inter-Regional Approach. Sustainability.

[40] Frenk J., Frejka T., Bobadilla J.L., Stern C., Lozano R., Sepúlveda J., José M. (1991). The epidemiologic transition in Latin America. Bol. Oficina Sanit Panam..

[41] Santosa A., Wall S., Fottrell E., Högberg U., Byass P. (2014). The development and experience of epidemiological transition theory over four decades: A systematic review. Glob. Health Action.

[42] Tyrovolas S. (2020). The burden of disease in Saudi Arabia 1990–2017: Results from the Global Burden of Disease Study 2017. Lancet Planet. Health.

[43] El Bcheraoui C., Mimche H., Miangotar Y., Varsha S.K., Ziegeweid F., Krohn J.K., Ekat M.H., Nansseu J.R., Dimbuene Z.T., Mokdad A.H. (2020). Burden of disease in francophone Africa, 1990–2017: A systematic analysis for the Global Burden of Disease Study 2017. Lancet Glob. Health.

[44] Robert W. (2008). La mortinatalité: Éclairage historique sur des problèmes persistants d’estimation et d’interprétation. Population.

[45] GBD 2015 Mortality and Causes of Death Collaborators (2016). Global, regional, and national life expectancy, all-cause mortality, and cause-specific mortality for 249 causes of death, 1980-2015: A systematic analysis for the Global Burden of Disease Study 2015. Lancet.

[46] Organisation Mondiale de la Santé (2004). Rapport sur la Santé Dans le Monde. https://www.who.int/whr/2004/fr/.

[47] Dixon S., Mc Donald S., Roberts J. (2002). The impact of HIV and AIDS on Africa’s economic development. BMJ.

[48] Boerma J.T., Nyamukapa C., Urassa M., Gregson S. (2003). Understanding the uneven spread of HIV within Africa: A comparative study of biologic, behavioral, and contextual factors in rural populations in Tanzania and Zimbabwe. Sex. Transm. Dis..

[49] Freeman T., Gesesew H.A., Bambra C., Giugliani E., Popay J., Sanders D., Macinko J., Musolino C., Baum F. (2020). Why do some countries do better or worse in life expectancy relative to income? An analysis of Brazil, Ethiopia, and the Unite States of America. Int. J. Equity Health.

[50] Shaw W.J., Horrace C.W., Vogel J.R. (2005). The Determinants of Life Expectancy: An Analysis of the OECD Health Data. South. Econ. J..

[51] Pająk A., Kozela M. (2011). Cardiovascular Disease in Central and East Europe. Public Health Rev..

[52] Moser K., Shkolnikov V., Leon D.A. (2005). World mortality 1950-2000: Divergence replaces convergence from the late 1980s. Bull World Health Organ..

[53] Meslé F., Vallin J. (2017). The End of East–West Divergence in European Life Expectancies? An Introduction to the Special Issue. Eur. J. Popul..

[54] Men T., Brennan P., Boffetta P., Zaridze D. (2003). Russian mortality trends for 1991-2001: Analysis by cause and region. BMJ Clin. Res..

[55] Doll R., Peto R., Hall E., Wheatley K., Gray R. (1994). Mortality in relation to consumption of alcohol: 13 years’ observations on male British doctors. BMJ.

[56] OECD (2020). States of Fragility.

[57] Muntele I., Istrate M., Bănică A., Horea-Șerban R.I. (2020). Trends in Life Expectancy in Romania between 1990 and 2018. A Territorial Analysis of its Determinants. Sustainability.

[58] Nick J., Menzies M., Radchenko D. (2021). Covid 19 s wawe in Europe and the United States. Chaos.

